# Using the School Environment to Promote Walking amongst Adolescent Females: A Mixed-Method Study

**DOI:** 10.3390/children6030049

**Published:** 2019-03-23

**Authors:** Angela Carlin, Marie H. Murphy, Alison M. Gallagher

**Affiliations:** 1 Centre for Exercise Medicine, Physical Activity and Health, Sports and Exercise Sciences Research Institute, University of Ulster, Jordanstown Campus, Newtownabbey BT37 0QB, UK; mh.murphy@ulster.ac.uk; 2 Nutrition Innovation Centre for Food and Health (NICHE), Biomedical Sciences Research Institute, University of Ulster, Coleraine Campus, Coleraine BT52 1SA, UK; am.gallagher@ulster.ac.uk

**Keywords:** physical activity, adolescents, walking, school environment

## Abstract

Schools have the potential to promote physical activity (PA) in adolescents through physical education (PE) and extra-curricular PA. The aims of this study were to firstly understand the experiences of adolescent females who participated in a school-based walking programme (the Walking In ScHools (WISH) study) and secondly, to assess the potential for schools to further promote PA outside of structured PE. A sample of female participants (*n* = 45, mean age 13.1 years) who participated in the WISH study were randomly selected to participate in focus group discussions, to explore their experiences of the intervention. In addition, an online survey was distributed to all post-primary schools (*n* = 208) in Northern Ireland to assess the provision of extra-curricular PA and further evaluate the feasibility of the WISH study. In total, six focus groups were conducted. Walking during the school day was viewed as an acceptable form of PA by adolescent females, providing an opportunity to be active with friends, and helped participants overcome barriers previously associated with being active at school. Responding schools (*n* = 59) identified adolescent females and non-sporty pupils as sub-groups who would benefit most from participation in a school-based walking programme. This study has highlighted that the delivery of a walking programme within the school setting is acceptable, warranted and practically feasible from the point of view of adolescent females and key stakeholders within the school setting.

## 1. Introduction

Physical activity (PA) is associated with numerous health benefits for children and adolescents [1], and children and young people (<18 years) are currently recommended to take part in at least 60 min, and up to several hours of moderate-to-vigorous PA (MVPA) per day [2]. Despite this, many young people are failing to meet current recommendations for PA [3,4]. Evidence also consistently demonstrates females are less likely to meet the PA recommendations compared with their male counterparts [3,4]. Schools represent a fertile environment for the promotion of PA behaviours; however, there is a lack of consensus on how best to promote PA within the school setting to ensure the maintenance of PA behaviours into late adolescence, and adult life [5]. Furthermore, whilst evidence has highlighted the importance of a whole-school approach to the promotion of PA, the onus for further promoting PA within the school environment generally falls to physical education (PE) teachers and the PE curriculum [6].

School-based PE provides an opportunity for young people to participate in structured, regular PA [7], and has the potential to contribute towards time spent in MVPA [7,8]. Time spent in PE has been shown to decrease as adolescent females’ progress through post-primary education [9]. This, coupled with curriculum squeezes on PE, means that other opportunities within the school setting may be increasingly important in promoting overall PA in this age group. Such opportunities may include increasing the provision of non-curricular PA at break and lunch-time (recess periods), or developing strategies to utilise timetabled PE [10], so that the school setting can be more effective in helping adolescent females meet the PA recommendations.

The provision of extra-curricular activities within school has been shown to increase PA, with active travel to school, summer camps and school recess identified as possible opportunities to promote PA outside of timetabled PE [11]. The contribution of school recess to total PA in young people has been shown to decline as children make the transition from primary to post-primary education [12], with females less likely to be active during recess [13,14]. The limited number of PA interventions targeted at school recess periods, particularly in adolescent females, limits conclusions on the effectiveness of such interventions on PA in children and adolescents [15]. Furthermore, the intervention components within these studies (playground markings and equipment) may be less feasible within the post-primary setting [16]. Evidence has highlighted that school-based interventions that target females only may be more effective than mixed-gender interventions at increasing PA [17].

Walking has been at the cornerstone of PA promotion in adults [18], and is recognised as a suitable means of PA promotion for adolescents also [19]. Brisk walking can contribute to their MVPA guidelines in children [20] and may overcome some frequently cited barriers to adolescent female PA participation at school (e.g., uniforms, not wanting to exercise in front of peers) and the challenges associated with changing priorities in this population [21]. Walking interventions have the potential to increase PA in children and adolescents [22]; however, to date, there are limited interventions in adolescent females [22]. Research examining the potential for walking to increase PA in adolescent females has received increased focus in recent years [16,22,23,24]; however, less is known about the acceptability of such interventions. When developing and implementing interventions, it is important that policy makers and practitioners actively consult with adolescent females to gain an understanding of what works in this age group and to identify facilitators and barriers to PA in this population, which can inform the design of future interventions [25], as well as engaging adolescent females in such interventions [17]. In addition, there is limited evidence on the acceptability of such interventions from a schools’ perspective, and further research is needed to understand how PA interventions, in particular walking, can meet the needs of schools and female students in terms of increasing opportunities for non-curricular PA.

The aim of this study was to examine the potential of using the school environment to promote walking behaviours among adolescent females. The study involved a mixed-methods approach to a) understand the experiences of adolescent females involved in a school-based walking programme, and factors that may influence their participation; and b) assess stakeholders’ (schools’) perceptions of implementing a walking programme in the target population.

## 2. Material and Methods

### 2.1. Phase 1—Focus Groups with Adolescent Females

#### 2.1.1. Sample Selection

This phase of the study aimed to assess the preferences of adolescent females for a school-based walking programme, and factors that may influence their participation. All pupils (*n* = 199) who consented to take part in the Walking In ScHools (WISH) study were eligible to participate in this follow-up study. Briefly, the WISH study was a 12-week, school-based randomised controlled trial that aimed to increase PA amongst adolescent females. Female pupils took part in structured walking sessions, of 15 min duration, which were delivered by older pupils during the school day. On completion of each walking session, participants received a reward stamp which could be accumulated and exchanged for small prizes and rewards. Further information on the WISH study and main findings have been summarised previously [16].

A random sample of participants (*n* = 45) were selected from all those who had previously participated in the WISH study (from both intervention and control groups), using a random number generator function. Focus groups were conducted two months after the final follow-up measurements of the WISH study were completed. Information on the focus group discussion and the role of participants with the group were provided before the focus group began, and all participants were reminded that they did not have to participate in the discussion if they did not want to. Written consent from parents/guardians and assent from participants was obtained for participation in the intervention and for this follow-up evaluation study. Verbal consent was obtained from all participants before the focus group discussion began. The study was conducted in accordance with the Declaration of Helsinki, and was approved by the University of Ulster Research Ethics Committee (REC/11/0236).

#### 2.1.2. Focus Groups

All focus group discussions followed a semi-structured discussion guide designed to explore some key components of the WISH study and were facilitated by the same moderator. Six focus groups were conducted in total, with the number of participants in each focus group ranging from 6–8 females. Participants in each focus group were from a mix of classes within each school; however, the majority of participants knew the other members of the focus group. The main focus of the discussions was centred on how to promote PA in adolescent females, with particular reference to the WISH study and acceptability, barriers and facilitators of the intervention in this target group (Table 1). At the beginning of the focus group discussions, the moderator provided a general definition of PA for participants, highlighting that PA referred to any movement, and was not limited to structured exercise, team sports or PE. Those who had completed the actual walking programme within schools (i.e., the intervention group) were asked to base responses on their experiences of being involved in the programme. Both groups were asked the same general questions; however, the control groups were provided with a brief overview of what the WISH study involved and asked to share their thoughts and ideas on how the intervention would be received by them or to offer suggestions to change the intervention.

All focus group discussions were conducted within the school environment, at a convenient time decided upon by school staff. Focus group discussions ranged from 18 min to 40 min in duration (average focus group discussion lasted 27 min).

#### 2.1.3. Data Analysis

All focus group discussions were audio-recorded, transcribed verbatim and analysed thematically, following a deductive approach [26]. Following familiarisation with the data, each transcript was reviewed for meaningful quotes and systematically coded by a member of the research team. Potentially relevant codes were grouped together to develop themes, which were reviewed to ensure representativeness. These themes were then reviewed by a member of the research team to ensure the themes were representative of the coded excerpts. Coding and reviewing of themes was repeated independently by a second member of the research team.

Names of participants or school staff mentioned during the discussion were removed from the transcripts to ensure confidentiality. Quotations from participants are used to highlight the development of the key themes, with the following coding applied to each quote: Intervention participants (INT); Control participants (CON).

### 2.2. Phase 2—Survey of Post-Primary Schools

#### 2.2.1. Sample Selection

All post-primary schools in Northern Ireland (*n* = 208; 140 secondary and 68 grammar schools; 157 co-educational, 24 male-only and 27 female-only) were eligible for participation in the survey. This study was approved by the School of Biomedical Sciences Filter Committee (FCBMS-14-092). All school principals were contacted via e-mail and provided with information on the study. Principals who agreed to their schools’ participation in the survey were then instructed to identify a suitable member of school staff, who was in frequent contact with female pupils aged 11–14 years, to complete the survey. Surveys were distributed and completed by schools in spring 2015, after the WISH study and follow-up focus groups (Phase 1 of the present study) had been completed. The research team specifically requested that the survey was not completed by staff within the PE department, as it was considered that PE teachers may be more inclined to present a more positive view of their school’s performance in relation to the provision of extra-curricular PA. Instead, schools were asked to identify non-PE members of staff who were familiar with the extra-curricular provision of PA within the school (herein referred to as the school representative).

#### 2.2.2. Survey

The survey was designed to assess stakeholders’ (schools) perceptions of implementing a walking programme in the target population (adolescent females). Within this, the survey examined the existing provision of extra-curricular activities within schools, the potential for schools to further promote PA outside of structured PE and the practical issues associated with implementing a school-based walking intervention for adolescent females. The survey was designed to address existing gaps in the literature in relation to the provision of extra-curricular activities in post-primary schools in Northern Ireland, and to gauge the potential of rolling out PA interventions, including walking interventions (i.e., the WISH study) to schools across Northern Ireland. A survey of post-primary schools in England was consulted when designing the pre-determined responses for schools to select [6].

The survey was accompanied with a cover letter providing instructions on completion of the survey, and also included a definition of extra-curricular activity to clarify for staff exactly what the survey was focusing on. The questionnaire was completed electronically by participating schools, via Survey Monkey (San Mateo, CA, USA). As an incentive, all schools that completed the questionnaire were entered into a draw to win one of three £50 vouchers to be spent on sports equipment for their school. All school names were replaced with a unique identification number following data collection. Additional demographic information on individual schools including school type, school management type, setting (urban/rural), enrolment data, gender breakdown, % entitled to free school meals and number of pupils with special education needs (SEN) was obtained from the Department of Education website [27].

#### 2.2.3. Data Analysis

Chi-square test for goodness of fit was used to compare the representativeness of the study sample to the overall number of post-primary schools in Northern Ireland. Descriptive statistics were used to compare school representative responses.

## 3. Results

### 3.1. Phase 1—Focus Groups with Adolescent Females

Participant characteristics: In total, 45 female pupils participated in the focus group discussions (*n* = 23 intervention; *n* = 22 control). The mean (SD) age of the participants was 13.11 (0.60) years. In total, six focus groups were completed (*n* = 3 intervention (INT) schools, *n* = 3 control (CON) schools). Saturation was achieved during focus group discussion 6 and no further focus groups were conducted. The focus group discussions generated a number of areas for discussion around evaluation of the WISH study and the potential for walking to promote PA within the school setting. The main themes are summarised below:

#### 3.1.1. Facilitators to Being More Active During the School Day

Participants discussed the importance of support and encouragement from friends in being more active: “Because they encourage you, erm they’re like there and talk to you and you don’t feel awkward around people you don’t know” (INT, Group 3). Peers also played an important role in influencing participants to be active, with some having a positive experience with classmates: “Yeah the girls that are sporty in our school they would never, they wouldn’t judge you like, like if you’re not good at something that’s just like ok.” (INT, Group 5). Some participants had negative experiences with peers when participating in PA, which deterred them from future participation: “… people that you know are judgemental and they’ll stay stuff about you afterwards.” (CON, Group 1).

When discussing the provision of activities to engage females in PA, the importance of offering activities that were fun was a key determinant in whether participants would want to participate or not: “I think definitely fun things, because it’s easier to enjoy it if it’s something, if you’re not really thinking about the fact that you’re doing exercise…” (CON, Group 1) and “Maybe go and do activity for the craic (*fun*), most people would like that.” (CON, Group 3). Participants noted that being active during the school day could overcome barriers associated with trying to be active outside of school time, including lack of time: “I get home pretty late because of the buses and I don’t have time, not enough time to fit everything in in the evening.” (INT, Group 5), and competition from other after-school activities: “Like, like the schoolwork’s one but then like stuff after school too, your own stuff, it can get in the way with afterschool activities and sometimes they clash and you have to like choose.” (CON, Group 4)

#### 3.1.2. Walking as a Form of Physical Activity

A number of advantages for walking as a form of PA were cited by adolescent females. The majority of participants who were in the intervention group enjoyed walking as it was not an overly strenuous form of activity (Table 2), while participants from the control group noted that walking was an acceptable form of activity that did not require any special skills (Table 2). Participants (intervention group) also enjoyed the social aspects of the walking group: “I found it like really good to go, like a way I could meet up with my friends from other classes and stuff, and it was a really good way to meet other people in our year.” (INT, Group 6). Similar advantages of walking within the school day were cited by participants in the control schools, in relation to the advantages of walking over other forms of activity: “It’s just like a wee club that you can like go and talk to your friends while you exercise so it’s like you don’t know you’re exercising so it’s just like a wee group, you’re like exercising without realising you’re exercising, you don’t really think of it that way.” (CON, Group 1). Participants from control schools also felt that it was a good way to engage less active pupils in PA, noting that walking is a non-competitive form of PA (Table 2).

#### 3.1.3. Intervention Components

Focus group participants discussed a number of the key intervention components, including the type of PA (walking), the role of the walk leaders and the use of incentives within the intervention. Participants who were in the intervention group noted that they enjoyed the opportunity to be active during the school day: “Like it, it kept you going, you weren’t just standing there or sitting down, you were walking… it doesn’t feel like you’re doing exercise.” (INT, Group 3) and described the intervention as an acceptable form of activity, that did not draw attention from peers (Table 2).

Participants from the control group, who had not taken part in the WISH intervention, noted that walking during the school day would provide an opportunity to overcome some of the previously cited barriers to being more active, for example, lunch-time activities only available for male pupils: “Most of the girls would sit down at lunch because they know, we know we can’t go and play football and run like the boys so it would be good for us to like say like go a walk or whatever.” (CON, Group 4).

Participants taking part in the walking intervention found the rewards they received in exchange for their participation to be a motivating factor in attending the walking sessions (Table 3). Those in the control schools placed less emphasis on the importance of the rewards: “It wouldn’t matter but like it’s nice to have a reward.” (CON, Group 4).

Some participants in the intervention schools felt the role of the walk leaders was unnecessary, with a number highlighting that the presence of the walk leaders could be off-putting at times (Table 3). Instead, some participants would have preferred to have their own classmates and peers take charge of leading the walks: “I think we’re pretty respectable of each other as a year… we wouldn’t like not listen to them.” (INT, Group 5). In addition, participants commented that the walk leaders weren’t helpful at setting the pace of the walks: “A lot of time they were just at the back of the group.” (INT, Group 6). However, participants still favoured having older pupils lead the walks as opposed to teachers or other school staff (Table 3).

### 3.2. Phase 2—Survey of Post-Primary Schools

#### 3.2.1. Demographics of Responding Schools

Of the 208 schools approached, a school representative from *n* = 59 post-primary schools completed the survey in full; there was a response rate of 28.4%. There was no significant differences in the proportion of schools for school type, gender breakdown or location (all *p* > 0.05), when comparing the responding schools to the overall Northern Ireland averages. The majority of responding schools were mixed gender (79.7%), and located within an urban setting (83.1%). The mean percentage of pupils entitled to free school meals in responding schools was 27.5%, while the proportion of pupils with SEN in responding schools was 24.2%.

#### 3.2.2. Provision of Extra-Curricular Activities

Based on responses from school representatives, all female-only schools and mixed-gender schools stated that they offered extra-curricular activities on a regular basis to female pupils aged 11–14 years. After-school was the most popular time for extra-curricular activities to be offered within schools (89.9% of responding school representatives). These activities were offered to a lesser extent at other times within the school day, including before school (6.8%), break-time (3.4%) and lunch-time (44.1%), and also at the weekends (5.1%). Specialist staff, i.e., PE teachers, were tasked with the implementation of extra-curricular activities across all responding schools. Non-specialist staff, i.e., non-PE teachers, were also responsible for the implementation in over half of responding schools (55.9%).

**Figure 1:**
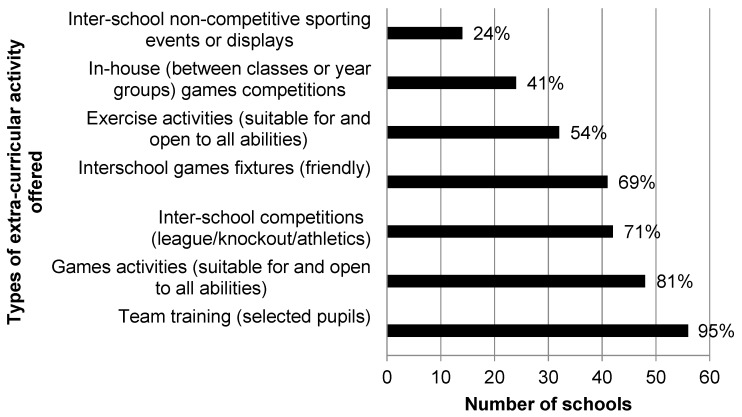
Extra-curricular activities offered within schools on a regular basis.

The types of extra-curricular activities offered within schools are highlighted in Figure 1. Team training, for selected pupils only, was the most commonly offered extra-curricular activity (94.9% of responding schools). Responding schools identified staffing as the main barrier to the provision of extra-curricular activities from a schools’ perspective, followed by time and interest from pupils. Schools identified dance classes/Zumba (40.7%), exercise classes/circuits (40%) and access to fitness suites/gyms (25.4%) as the three activities they would like to see offered within schools to encourage the uptake of more extra-curricular sporting options amongst pupils. All responding schools had established links with at least one outside agency in efforts to further promote PA outside of timetabled PE (Table 4). 

#### 3.2.3. Influences on Adolescent Physical Activity from the Schools’ Perspective

**Figure 2:**
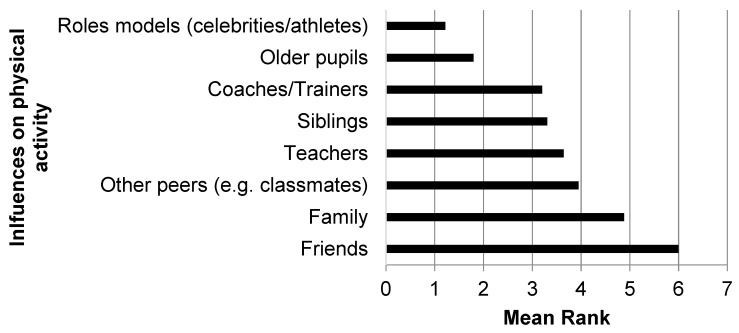
Main influences ^†^ on physical activity in younger female pupils (11–14 years) from a schools’ perspective. ^†^ Responding schools asked to rank the main influences from 1 to 8 (where 1 is the least influential and 8 is the most influential).

**Figure 3:**
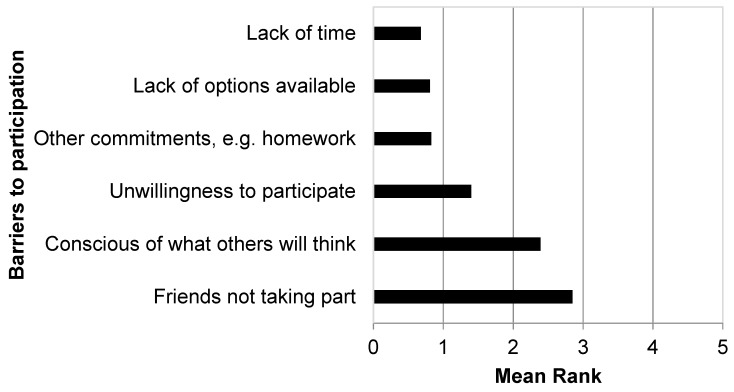
Perceived main barriers ^†^ to participation in extra-curricular physical activity from adolescent females (aged 11–14 years). ^†^ Responding schools asked to rank the main barriers from an adolescent females’ perspective from 1 to 6 (where 1 is the least and 6 is the greatest barrier).

Responding schools identified friends as the greatest influence on PA in younger female pupils aged 11–14 years (Figure 2), followed by family. Role models (celebrities/athletes) were identified as the least influential, followed by older pupils. Friends not taking part was noted as the greatest barrier to participation in extra-curricular PA from an adolescent females’ (aged 11–14 years) perspective (Figure 3), with responding schools selecting lack of time as the least important barrier from an adolescent females’ perspective.

#### 3.2.4. Perceptions of Adolescent Physical Activity Participation

The majority of responding schools (59.3%) highlighted that they felt PA levels differed between male and female pupils aged 11–14 years. The main reasons for these differences were attributed to boys participating in more structured PA (13.6%), boys being more active at break and lunch-time (8.4%) and more clubs on offer for boys (10.2%). The three sub-groups most frequently identified by schools that would benefit most from increased participation in PA included females aged 11–16 years (66.1%), females aged 16+ years (32.2%) and ‘non-sporty’ pupils/ those who dislike participating in competitive sports (30.5%).

#### 3.2.5. Feasibility of the WISH Study within the School Setting

Of the 59 schools surveyed, just under one-fifth currently offered any types of walking intervention, which may impact upon the resources needed to facilitate the WISH intervention as part of the provision of activities to pupils. In terms of walking as a means of increasing PA, 58.3% of schools agreed walking would be an effective means of promoting PA within the school day. The majority of responding schools highlighted that other teachers, i.e., not PE teachers, would be best placed to deliver a walking intervention within the school day (67.8%). Other suitable facilitators for a walking programme included older pupils trained as walk leaders (49% of responding schools) and PE teachers (30.5% of responding schools).

Schools were asked to identify three main issues that would be problematic when implementing a walking intervention within the school environment. Open-ended responses were grouped together and coded; the main issues identified by schools included safety (64.9%), supervision and suitable ratios of staff to pupils (44.1%), time (44.1%) and lack of suitable environments for walks (18.6%). Female pupils aged 11–16 years (40.7%), older female pupils (42.4%) and those pupils who were ‘non-sporty’ (23.7%) were identified by responding schools as the sub-groups within schools that participating in a school-based walking intervention would most appeal to. Schools identified friends also taking part (54.2% of responding schools) as the factor that would most encourage younger pupils to participate in a school-based walking intervention.

## 4. Discussion

The aim of this study was to examine the experiences of adolescent females involved in a school-based walking intervention, and the potential of using the school environment to promote walking behaviours among adolescent females, from the point of view of the target population (adolescent females) and key stakeholders (school representatives). Phase 1 of the study identified walking as an acceptable form of PA to participate within the school day from the perspective of the target population (adolescent females) and demonstrated the importance of social support in encouraging adolescent females to be more active. Phase 2 of the study highlighted that schools recognise the need to target adolescent females when promoting PA, and emphasised a number of practical issues that need to be considered when promoting PA (walking) within the school setting.

Based on qualitative data from the present study, adolescent females perceived walking to be an acceptable form of PA during the school day. Respondents noted that walking was a non-competitive activity that could be completed routinely within the school day without any specialist skills or competencies. Furthermore, the responses highlighted that walking could overcome some previously cited barriers to PA at school, including not wanting to wear a PE uniform or feeling conscious in front of peers [21]. The perceived ability amongst adolescent females has been identified as a barrier to participation in lunchtime PA [28]; therefore, presenting opportunities for adolescent females to walk during the school day provides an ideal opportunity to engage this group in PA that requires no skill/competency and that does not involve competition [29]. Evidence suggests that such activities are likely to be more acceptable to adolescent females [29,30]. A recent study on the acceptability of walking breaks during the school day reported 95% of students were satisfied with walking as a form of PA during the school day [24]; however, this study included both males and females. Although further research is needed to fully explain why males are more active during school recess [31], the differences in school time PA between genders is likely to be associated with gender roles [32]. Therefore, overcoming the identified barriers to PA and providing activities that are acceptable such as walking during the school day is of particular importance for PA promotion in adolescent females.

Over half of the responding schools agreed that walking would be an effective means of promoting PA within the school day, indicating that walking interventions may also be acceptable from a stakeholder (schools) point of view. A number of practical issues to implementation were identified from a schools’ perspective, including safety, supervision and a lack of suitable environments for walks. Walking interventions have previously been shown to be practically acceptable from a schools’ point of view, placing few organisational demands on teachers [24]. Addressing the identified barriers from schools is important in future intervention delivery and development, as such barriers may have an impact on how interventions are implemented and delivered in the real-life school setting.

Research has previously identified factors such as supporting teacher autonomy [33], time and a supportive school environment [34] as key indicators of successful intervention implementation in the school setting. The use of older pupils (peer leaders) to facilitate the walking sessions within the WISH study was novel [16], and may provide a valuable resource to overcome barriers such as staffing issues time constraints within schools. Approximately half of responding schools indicated that pupils, trained as walk leaders would be suitably placed to help deliver walking-based interventions within the school setting. Participants involved in the WISH study had mixed opinions on the role of the walk leaders, with some highlighting that they could organise and facilitate the walks themselves, without the need for older peers, while others felt it was important to have older peers present to ensure that the walks were conducted properly. Identifying ways to increase social support for PA, particularly from peers, should be a priority for schools when trying to promote PA during school recess [35], for example, through peer mentoring schemes [28,29,36].

The role of friends in providing support and encouragement was key in helping promote PA from the adolescent females’ perspective. A number of participants (intervention group) highlighted that peers could provide positive support while others in the control arm suggested that peers may have a negative influence on participation in PA. School staff also identified friends as the main influence on adolescent females’ willingness to take part in walking interventions at school. The involvement of friends and peers is directly associated with PA amongst adolescents [37,38], and can influence PA both positively and negatively [38]. Peer support, peer acceptance and the presence of friends/peers are all positively associated with PA in adolescents and should be considered in interventions targeted at this population [38]. Given that the WISH study was the first intervention to explore the use of older peer leaders to promote walking behaviours, further research is needed to understand the relationship between peers with regards to walking.

Providing PA opportunities that enable adolescent females to socialise with their friends was identified from focus group discussions as a key component within the WISH intervention. Giving adolescent females a peer network to engage in PA with during the school day can overcome the barrier of having no-one to be active with, which has been previously cited as preventing adolescents from being active during school recess [39]. Given that enjoyment [40,41] and opportunities to socialise with peers [40] are strongly correlated with PA participation in adolescent females, the positive experiences of participants within this intervention in relation to enjoyment and socialising should be highlighted when planning recruitment strategies for future school-based walking programmes [42].

The tendency for extra-curricular PA to reflect the content of timetabled PE is well documented within the literature [6,43], and was reflected within the present study. Team training sessions for selected players, games activities and inter-school matches were the most common extra-curricular activities offered within responding schools on a regular basis, which is consistent with previous research in post-primary schools [6]. With the exception of games activities for all pupils, the extra-curricular activities commonly offered by schools within the present study were all team-based, competitive sports for a select group of pupils, usually the most skilled. Placing an emphasis on such sports within the provision of extra-curricular PA can mean that many students who would benefit from participation in additional PA are excluded [25]. Providing fun, non-traditional activities in an environment where young people felt comfortable has been identified as a strategy to engage adolescent females in PA [21]. Thus, further approaches to providing non-competitive, informal opportunities for PA such as walking should be explored in this target population.

## 5. Strengths and Limitations of the Study

This study has provided insight into the acceptability of walking and, specifically, a school-based brisk walking programme (the WISH study) both from the perspective of participants involved in the study and from the perspective of post-primary schools who may implement similar interventions in their provision of extra-curricular PA during the school day. The rich, qualitative data generated from the focus group discussions provide detailed insight into the experiences of adolescent females involved in a school-based walking intervention. The survey targeted at staff in post-primary schools is the first to provide detailed information on the provision of non-curricular PA within schools in Northern Ireland, and has highlighted the barriers currently facing schools in relation to the provision of extra-curricular PA. Schools represent a key stakeholder in terms of the provision of PA to adolescents and this survey has provided insight into the potential for schools to further promote PA outside of PE. Comparisons between the responding schools in Phase 2 of the study (survey) and the Northern Ireland average shows that the study sample was representative of the wider post-primary school setting. Furthermore, non-PE members of staff, who were familiar with the schools extra-curricular provision of PA, were asked to complete the questionnaire, to reduce the risk of bias in over-reporting the current provision of extra-curricular PA.

Participants from the WISH study were randomly selected to participate in the focus group discussions to evaluate the intervention; therefore, the views expressed by those selected may not be representative of all participants who participated in the study. In addition, it is possible that participants may not have been completely honest when discussing certain aspects of the intervention, as they wanted to present themselves in a positive light to the researcher. Participants were familiar with the focus group facilitator from previous data collection, which may also have impacted upon the validity of their responses. The response rate from post-primary schools was low (28.4%) despite the use of incentives to promote responses. A lack of information on the response rates of schools to surveys limits comparisons [44]; however, the response rate within the present survey was comparable with surveys of school staff in relation to other health-related behaviours, for example, the emotional health of adolescents [45] and online-based surveys conducted in other educational establishments [46].

## 6. Conclusions

This study has highlighted that the delivery of a walking programme within the school setting is practical and acceptable, from the view of both students and school staff. Walking represents a suitable form of PA to be offered within the school setting, and overcomes some of the frequently cited barriers to PA from an adolescent female perspective. Future interventions within schools, involving opportunities to participate in PA outside of timetabled PE, should be targeted at the identified sub-groups who would benefit most from increased participation, for example, non-sporty pupils and adolescent females. Incorporating strategies such as social support from friends and peer mentoring may be effective in promoting PA during the school day. The positive experiences identified in this study should be reinforced when recruiting adolescent females onto future walking interventions, and the barriers identified by schools in relation to safety and staffing concerns should be addressed.

## Figures and Tables

**Table 1:** Semi-structured discussion guide for focus groups.

Focus Groups
What are your favourite types of physical activity?
What factors influence your own physical activity? What people? What are the barriers towards your participation in physical activity?
What factors or approaches would encourage you to participate, or participate more, in physical activity?
Think back to when you first received information about this study, what encouraged you to sign up or take part in the study?
What do you think schools could do to help people your age become more active?
What do you think about the walking intervention and its components, for example, walking during school, walk leaders, rewards?
Are there any other thoughts or ideas you would like to share that haven’t previously been covered in today’s discussion?

Warm up questions to get participants familiar with the focus group environment.

**Table 2:** Advantages of walking from thematic analysis.

Advantages of Walking	Quote
Not a ‘sport’	“I liked it because it wasn’t anything too strenuous, ‘cause we can’t really do much, cause like we wear a skirt…” (INT, Group 5)
	“It doesn’t require too much energy, you don’t actually have to be good at sport to walk” (INT, Group 6)
	“It doesn’t involve any skill or anything, if you’re not good you can still do it.” (CON, Group 1)
	“You don’t realise you’re doing it, like when you’re in your PE kit and you’re running about and your teacher’s shouting at you to run faster you’re like aw I hate doing this but you know when you’re walking you’re like doing it every day so you can do it at your own pace.” (CON, Group 1)
Acceptable form of activity	“Again its noticeable if you’re going, if you were to go out at lunch and run about the school you’d be a bit… everyone would be a wee bit like what are they doing but whenever it’s just walking, because everyone walks anyway so no one passes any remarks… we weren’t embarrassed walking around.” (INT, Group 5)
	“Because you can’t get hurt, you can do it at your own pace.” (CON, Group 2)
	“It doesn’t involve any skill or anything, if you’re not good you can still do it.” (CON, Group 1)
Not competitive	“It’s not like if you’re running or something you might be really slow, and there’s other really sporty people that you think might judge you at it, like you’re all walking, it’s just walking.” (CON, Group 1)
	“People can stay at the same pace without being competitive with everybody.” (CON, Group 2)

Quotes presented are examples of quotes obtained from the focus groups discussions. CON: control, INT: intervention, PE: physical education.

**Table 3:** Intervention characteristics based on thematic analysis.

Intervention Component	Quote
Opportunity for socialising	“I found it like really good to go, like a way I could meet up with my friends from other classes and stuff, and it was a really good way to meet other people in our year.” (INT, Group 6)
	“It’s just like a wee (small) club that you can like go and talk to your friends while you exercise so it’s like you don’t know you’re exercising so it’s just like a wee (small) group, you’re like exercising without realising you’re exercising, you don’t really think of it that way.” (CON, Group 1)
Rewards	“Yeah, you’re getting a reward for doing it, it encouraged people to go.” (INT, Group 5)
	“Making sure you do it.” (INT, Group 3)
	“It makes you feel better for what you’ve done… feels like you’ve actually did something.” (INT, Group 3)
Walk Leaders	“I think that erm, when the leaders were like shouting at and giving you like, it made you feel under pressure to walk faster and you didn’t get like a proper pace.” (INT, Group 3)
	“Yeah like at least whenever were not with teachers and stuff it just looks like we’re going for a bit of a dander (short walk) and having a bit of craic (fun).” (INT, Group 5)
	“You’d have to get along with them though, you wouldn’t want to walk with someone that you didn’t like, you wouldn’t want to be near somebody you didn’t like.” (CON, Group 1)

Quotes presented are examples of quotes obtained from the focus groups discussions. CON: control, INT: intervention.

**Table 4:** Number (%) of responding schools who have established links with outside agencies to promote physical activity outside of timetabled physical education.

Outside Link	Number of Responding Schools	Percentage of Responding Schools
Qualified/professional coaches	50	84.7
Local sports clubs	48	81.4
Local sports centres	37	62.7
Sports development officers	35	59.3
National governing bodies	29	49.2
Exercise leaders/instructors	29	49.2
Regional sports council	28	47.5
PE advisers/advisory teachers	21	35.6
Local health promotion unit/officers	17	28.8
School sports association	14	23.7
Other schools/further education colleges	13	22.0
Universities	13	22.0
**Other ^†^**	2	3.4

^†^ Placement students, teacher volunteers.
